# Palliative and end-of-life nursing care in Saudi Arabia: A systematic review of nursing practices, challenges, and patient–family outcomes

**DOI:** 10.1017/S147895152610203X

**Published:** 2026-03-06

**Authors:** Ateya Megahed Ibrahim, Donia Elsaid Fathi Zaghamir

**Affiliations:** College of Nursing, Prince Sattam Bin Abdulaziz University, Alkharj, Saudi Arabia

**Keywords:** Palliative care, end-of-life care, nursing, Saudi Arabia, communication, spiritual care, cultural influences, home-based palliative care

## Abstract

**Background:**

Palliative and end-of-life (EOL) care is gaining increasing importance in Saudi Arabia due to the rising burden of chronic and life-limiting illnesses. Nurses play a central role in delivering comprehensive, culturally appropriate palliative care; however, their practices are influenced by educational preparation, institutional support, and sociocultural and religious contexts. To date, evidence on palliative nursing care in Saudi Arabia remains fragmented and insufficiently synthesized.

**Aim:**

This systematic review aimed to synthesize existing evidence on palliative and EOL nursing care in Saudi Arabia, with a focus on nursing practices, challenges, cultural and spiritual influences, and patient and family outcomes.

**Methods:**

A systematic literature search was conducted in January 2025 using PubMed, Scopus, CINAHL, Web of Science, Google Scholar, and Saudi grey literature sources. Empirical qualitative, quantitative, and mixed-methods studies addressing palliative or EOL nursing care in Saudi Arabia were included. Study selection followed PRISMA guidelines, and methodological quality was appraised using appropriate critical appraisal tools. A narrative thematic synthesis was undertaken due to heterogeneity among studies.

**Results:**

Fourteen studies met the inclusion criteria. Findings indicated that nurses are actively involved in symptom management, therapeutic communication, psychosocial support, spiritual care, and family-centered care. However, substantial barriers were identified, including gaps in knowledge and training, limited formal palliative education, emotional burden, ethical challenges related to nondisclosure, and inconsistent institutional policies. Cultural and religious norms strongly influenced communication practices and decision-making processes. Studies also showed that structured palliative care services, particularly home-based and multidisciplinary programs, were associated with improved patient comfort, dignity, and family satisfaction, although access to such services varied across regions.

**Conclusion:**

Palliative and EOL nursing care in Saudi Arabia demonstrates commitment and potential but is constrained by educational, emotional, cultural, and systemic challenges. Strengthening nursing education, enhancing culturally sensitive communication and spiritual care training, expanding home-based palliative services, and providing institutional support for nurses’ emotional well-being are essential to improving the quality and equity of palliative care nationwide.

## Introduction

Palliative and end-of-life (EOL) care in Saudi Arabia is becoming a critically important component of the national healthcare agenda as the burden of chronic, life-limiting illnesses continues to grow (Alshammary et al. 2024). In recent years, advances in medical treatment, increased life expectancy, and the rising prevalence of non-communicable diseases such as cancer, heart failure, and chronic renal disease have intensified the demand for comprehensive and humane care for individuals nearing the end of life (Alosaimi et al. 2025; Zaidan 2025).

Although the Ministry of Health and tertiary hospitals have made notable strides in developing specialized palliative care units, substantial variability in service provision persists across regions, with many areas remaining underserved (Badreldin et al. 2025). Within this context, nurses play a central role in delivering palliative and EOL care, encompassing symptom and pain management, psychosocial and spiritual support, coordination of multidisciplinary care, and communication with patients and families (Alswaid et al. 2024; Shareifi et al. 2024). Despite this pivotal role, numerous studies have documented significant gaps in nurses’ knowledge and preparedness. For example, a cross-sectional survey of 154 Saudi nursing students reported very low average scores on the Palliative Care Quiz for Nursing (PCQN), indicating widespread misconceptions related to palliative care principles, pain management, and EOL practices (Khraisat et al. 2017).

Similarly, more recent research assessing critical-care nurses’ knowledge of palliative care in Saudi Arabia revealed poor overall understanding, particularly regarding the application of palliative principles within intensive care settings (Mitwalli et al. 2024). These educational gaps are further compounded by complex sociocultural and religious and spiritual dynamics (Bullock 2025). International nurses working in Saudi intensive care units have described the emotional strain and cultural mediation required to provide EOL care that respects Islamic traditions while maintaining professional standards (Mani 2024). Systematic reviews of nurse–patient communication in Saudi Arabia also highlight persistent barriers, including language differences, cultural misunderstandings, and religious considerations, particularly within a multicultural nursing workforce (Alshammari et al. 2019).

Despite these challenges, evidence from settings where structured palliative care services are operational suggests meaningful benefits for patients and families, including improved comfort, preservation of dignity, and greater satisfaction with care. However, because no comprehensive synthesis has focused specifically on the nursing dimension of palliative and EOL care in Saudi Arabia, a clear evidence gap remains. Addressing this gap through a systematic review is essential to integrate existing findings on nursing practices, barriers, and outcomes, and to inform policy, education, and clinical practice aimed at developing a culturally responsive and well-supported palliative nursing workforce.

## Methods

### Search strategy and data sources

A systematic literature search was conducted in January 2025 across multiple databases, including PubMed, Scopus, CINAHL, Web of Science, and Google Scholar, as well as Saudi grey literature through the Saudi Digital Library and Ministry of Health reports. Search terms included “palliative care,” “end-of-life care,” “nursing,” “Saudi Arabia,” “home-based care,” “spiritual care,” “communication,” and “symptom management,” combined with Boolean operators to capture relevant studies. The strategy was intentionally broad to include both academic and practice-oriented literature, ensuring representation from diverse healthcare settings such as hospitals, home health care, and academic dissertations. Studies published in English and Arabic were included, with no date restrictions to capture the full scope of research in the Saudi context.

### Review protocol and reporting standards

This systematic review was conducted in accordance with the Preferred Reporting Items for Systematic Reviews and Meta-Analyses (PRISMA 2020) guidelines. Although the review protocol was not prospectively registered in PROSPERO or a similar registry, a structured protocol was developed a priori to guide the review process. This protocol specified the review objectives, eligibility criteria, search strategy, study selection procedures, quality appraisal methods, and data synthesis approach. To enhance methodological transparency and reduce bias, study screening, full-text assessment, and quality appraisal were conducted independently by 2 reviewers, with disagreements resolved through discussion and consultation with a third reviewer when necessary.

### Eligibility criteria

This review included empirical qualitative, quantitative, and mixed-methods studies focusing on palliative or EOL nursing care in Saudi Arabia. In addition, selected nonempirical sources, such as national workforce reports, policy analyses, and expert commentaries, were included for contextual purposes only to support the interpretation of empirical findings and to describe the broader organizational and policy landscape of palliative care in Saudi Arabia. Nonempirical sources were not used as primary evidence in the thematic synthesis. Studies were excluded if they lacked relevance to nursing practice, focused exclusively on pediatric palliative care (as the scope of this review is limited to adult care to ensure consistency in practice, cultural, and ethical considerations), or did not address the Saudi context.

### Study selection

After removing duplicates using EndNote, 2 reviewers independently screened titles and abstracts to identify potentially relevant publications. Full texts of studies that passed the initial screening were then retrieved and assessed against the predefined inclusion and exclusion criteria. Inter-reviewer agreement for title and abstract screening was 90%, and any discrepancies were resolved through discussion, with a third reviewer consulted when consensus could not be reached. The screening process adhered to the PRISMA 2020 framework, ensuring transparency, reproducibility, and methodological rigor.

### Quality appraisal

The methodological quality of included studies was assessed using design-specific appraisal tools. The Critical Appraisal Skills Programme checklist was applied to qualitative research, the AXIS tool was used for cross-sectional surveys, and a combined qualitative–quantitative appraisal approach was employed for mixed-methods studies. Key domains evaluated included clarity of study aims, appropriateness of the sampling strategy, rigor of data collection, transparency of analysis, consideration of ethical issues, and thorough reporting of study limitations. Quality scores and key assessments for each study were summarized in the data extraction table to ensure transparency and provide an overview of methodological rigor. The results of this appraisal were also used to inform the synthesis of findings, giving greater weight to evidence from higher-quality studies and interpreting results from lower-quality studies with caution. This approach ensured that the conclusions drawn in the review were grounded in methodologically robust evidence while acknowledging limitations where appropriate.

### Data extraction

A standardized data-extraction form was used to record:

- Author(s) and year
- Setting and sample characteristics (nurses, patients, families)
- Study design and main focus
- Key findings and outcomes
- Reported challenges or barriers
- Quality appraisal score.

Extraction was piloted on a subset of studies and reviewed as a team to ensure consistency and accuracy.

### Synthesis of findings

Due to the heterogeneity in study designs, settings, and reported outcomes, a narrative thematic synthesis was employed to integrate the evidence from the included studies. Key findings from each study were systematically coded, and these codes were subsequently grouped into broader conceptual themes encompassing palliative nursing practices, barriers and challenges, cultural dynamics, emotional burden and coping strategies, and patient and family outcomes. These themes were then interpreted to identify common patterns, tensions, and areas of opportunity, with the aim of informing nursing practice, improving the quality of palliative care, and guiding policy development in Saudi Arabia. Findings from nonempirical sources were used solely to contextualize and support the discussion of systemic and policy-level issues and were not included in the primary thematic coding of empirical results.

### PRISMA flow diagram

**Figure 1.**
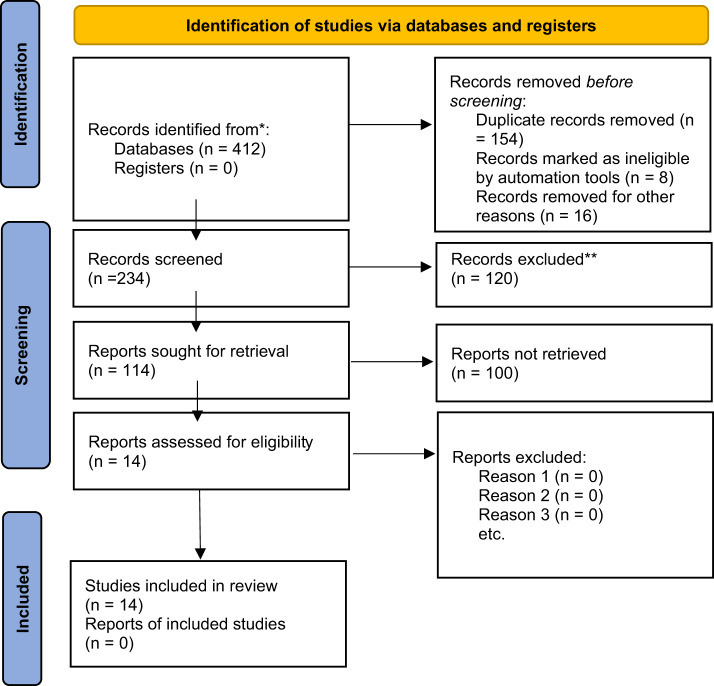
PRISMA 2020 flow diagram summarizing the identification, screening, eligibility assessment, and inclusion of studies in the systematic review.

A total of 412 records were identified through database searching (PubMed, Scopus, CINAHL, Web of Science, Google Scholar, and Saudi grey literature sources). Prior to screening, 154 duplicate records were removed. An additional 24 records were excluded before screening (8 records identified as ineligible by automation tools and 16 records removed for other reasons, such as clearly irrelevant document types). This resulted in 234 records being screened by title and abstract. Of these, 120 records were excluded because they did not meet the inclusion criteria. The remaining 114 reports were sought for full-text retrieval. Of these, 100 reports could not be retrieved (e.g., unavailable full text, inaccessible theses or institutional reports, or broken database links). Consequently, 14 full-text articles were assessed for eligibility. All 14 articles met the inclusion criteria and were included in the final review. No full-text articles were excluded after eligibility assessment (Figure 1).

### Methodological quality of included studies

The methodological quality of the included studies ranged from moderate to high. Qualitative studies generally demonstrated clear research aims, appropriate study designs, and coherent analytic approaches; however, several lacked detailed reflexivity statements or explicit discussion of researcher–participant relationships. Cross-sectional studies commonly met criteria related to clarity of objectives and reporting of results but were limited by convenience sampling, self-reported measures, and insufficient justification of sample size. Mixed-methods studies showed reasonable integration of qualitative and quantitative components, although the rationale for methodological integration was not always explicitly articulated. Across study designs, the most frequently identified methodological limitations included potential selection bias, limited generalizability, and reliance on self-reported data. Despite these limitations, all included studies were considered sufficiently rigorous to contribute meaningfully to the narrative synthesis. A summary of quality appraisal outcomes is provided in the data extraction table.

## Results

### Knowledge and competency in palliative nursing care

The review of 14 studies demonstrates a complex landscape of palliative and EOL nursing care across Saudi Arabia, highlighting both progress and persistent challenges. Across diverse healthcare settings, including intensive care units, oncology wards, home-health services, and administrative or leadership roles, nurses consistently exhibit gaps in core knowledge and competencies related to palliative care. Assessment tools, such as the PCQN, revealed low levels of understanding among both student and practicing nurses, with misconceptions around pain management, ethical decision-making, and spiritual care. Critical care nurses, while showing some familiarity with psychological and spiritual aspects of care, often lacked a comprehensive understanding of palliative principles, indicating an urgent need for focused education and professional development.

### Communication challenges and ethical tensions

Communication emerged as a major theme, with nurses frequently reporting difficulties navigating EOL conversations and decision-making processes in a context heavily influenced by Islamic values and collective family decision-making. Challenges were particularly pronounced in intensive care settings, where nurses needed to balance family preferences, patient autonomy, and institutional protocols. Nurses described emotional strain when delivering sensitive prognostic information, managing conflicts between family expectations and clinical recommendations, and maintaining compassionate interactions in high-pressure environments. These communication challenges were often compounded by systemic barriers, including the absence of specialized palliative teams, variability in home-care infrastructure, and lack of standardized institutional policies.

### Emotional burden and coping among nurses

The emotional burden on nurses was consistently documented across studies. High-acuity environments, such as ICUs, presented frequent exposure to death and suffering, which contributed to grief, moral distress, emotional exhaustion, and compassion fatigue. The absence of formal psychological support systems, such as structured debriefing sessions or counselling services, forced nurses to manage their emotional responses individually, often leading to sustained stress and potential burnout. Nurses highlighted the moral tension of providing care under conditions of limited resources, insufficient training, and high expectations for family-centered support.

### Cultural and spiritual dimensions of care

Cultural and spiritual aspects were central to the provision of palliative care in Saudi Arabia. Nurses routinely engaged in activities such as facilitating prayer, reciting religious texts, and liaising with Islamic leaders to support patients and families. However, many nurses expressed discomfort or reported a lack of formal training in spiritual care, leading to reliance on informal practices. Spiritual care was recognized as vital to patient and family well-being, yet the structured integration of spiritual support into clinical routines remained inconsistent.

### Patient and family outcomes and service availability

Patient and family outcomes appeared closely linked to the presence of structured palliative care services. Studies indicated that home-based care programs and specialized palliative teams resulted in improved symptom management, better coordination of care, and higher family satisfaction. Nevertheless, the availability of such services was uneven across regions, leaving many families without access to comprehensive palliative support. These disparities highlight inequities in care delivery and underscore the importance of expanding service coverage.

### Systemic and institutional barriers

Finally, systemic and institutional barriers significantly constrained the delivery of high-quality palliative nursing care. Workforce shortages, lack of standardized protocols, limited home-based care resources, and insufficient training pathways were recurrent challenges. The composition of the nursing workforce, including expatriate nurses with limited familiarity with local cultural and religious norms, further complicated effective communication and culturally sensitive care provision. These systemic limitations hindered the scalability and consistency of palliative care across the Kingdom, underscoring the need for coordinated strategies at both institutional and national levels (Table 1).

**Table 1.** Summary of included studies

No.	Author(s) and year	Study design	Setting/sample	Focus/main aim	Key findings/outcomes	Challenges/barriers
1	Alanazi 2024	Qualitative	ICU nurses, Saudi Arabia	Explore ICU nurses’ experiences in EOL care	Nurses experienced moral distress and emotional burden; emphasized the importance of communication and cultural sensitivity	Emotional stress, communication dilemmas, limited training
2	Almulla et al. 2024	Cross-sectional	Home care nurses, Saudi Arabia	Assess knowledge and practices of home-based palliative care	Moderate knowledge; symptom management and family support common; gaps in spiritual care	Limited training, inconsistent protocols
3	Mitwalli et al. 2024	Cross-sectional	Critical care nurses, Saudi Arabia	Assess knowledge regarding palliative care	Overall knowledge inadequate; some understanding of psychological/spiritual care	Knowledge gaps, workload pressures
4	Almobarak 2024	Nonempirical (contextual source)	Saudi hospitals	Discuss palliative care access and service expansion	Highlighted need for workforce expansion and integrated palliative services	Unequal distribution of services, limited access in rural areas
5	Mani 2024	Qualitative	International ICU charge nurses	Explore experiences providing EOL care in Saudi Arabia	Nurses had to bridge cultural gaps; emphasized the importance of spiritual and family-centered care	Cultural/language barriers, ethical dilemmas
6	Alodhialah and Almutairi 2025	Qualitative	Older adult patients and families	Explore access to palliative care and barriers	Limited awareness among patients/families; nurses key in facilitating care	System-level barriers, limited home-based options
7	Alqahtany et al. 2025	Nonempirical (contextual source: workforce report)	National-level	Describe palliative care physician and nursing workforce	Highlighted shortage of specialized nurses and uneven geographic coverage	Workforce gaps, lack of training programs
8	Alshammary et al. 2024	Review/survey	MOH hospitals	Update on palliative services, access, integration	Palliative services developing; patients/families report improved satisfaction where available	Uneven integration, limited resources
9	Al-Hindi and Dewan 2025	Qualitative	Palliative care nurses	Explore challenges in caring for terminally ill cancer patients	Emotional burden, communication with families, need for training	Emotional stress, lack of formal support systems
10	Alsolami et al. 2023	Cross-sectional	Nurses	Assess knowledge and attitudes toward palliative care	Moderate knowledge; misconceptions in pain management and EOL decision-making	Knowledge gaps, limited palliative education
11	Alshammari et al. 2023	Mixed-methods	Registered nurses	Explore beliefs about EOL care	Nurses emphasized compassion and family support; gaps in spiritual care	Training gaps, cultural communication challenges
12	Alodhialah et al. 2024	Qualitative	Nurses in Riyadh	Examine emotional resilience and coping	Nurses developed coping strategies; emotional burden significant	Lack of structured emotional support, moral distress
13	Callaghan et al. 2024	Cross-sectional	Family caregivers, tertiary care	Assess perceptions of symptom burden and satisfaction	Families satisfied with coordinated palliative services; symptom control improved	Limited availability of specialized services
14	Alrimali and Alreshidi 2024	Cross-sectional	ICU nurses, Saudi Arabia	Evaluate ICU nurses’ education, practice, and competence in palliative and EOL care	Only ∼30% had in-service training; training linked to higher competence scores	Limited training, authority to communicate, job satisfaction issues

## Discussion

This systematic review highlights a multifaceted and evolving palliative nursing landscape in Saudi Arabia, where nurses are central to EOL care yet face persistent challenges that constrain care quality. Across studies, knowledge gaps among nurses emerged as a critical barrier, with deficiencies in pain management, ethical decision-making, and spiritual care undermining the delivery of comprehensive palliative services (Alsolami et al. 2023; Mitwalli et al. 2024). These gaps suggest that current nursing curricula and professional development programs may not adequately prepare nurses for the complexities of palliative care, particularly in high-acuity settings where clinical and ethical decisions are frequent and demanding (Almulla et al. 2024). Addressing these educational shortcomings through structured curricula, competency assessments, and ongoing in-service training is essential for improving palliative nursing competence.

Communication challenges are both technical and cultural. Saudi cultural norms emphasize family-centered decision-making and may limit disclosure of prognostic information, creating ethical tension for nurses tasked with delivering patient-centered care (Mani 2024; Al-Hindi and Dewan 2025). Nurses’ experiences indicate that communication is not merely a technical skill but also a moral and cultural competency, requiring strategies to navigate family dynamics, religious expectations, and institutional policies. Training programs emphasizing cultural competence, ethical reasoning, and structured communication frameworks are necessary to equip nurses with the skills to manage these complex interactions effectively (Callaghan et al. 2024).

The emotional burden on nurses was consistently highlighted across studies, with grief, moral distress, and burnout described as pervasive issues, particularly in intensive care and high-acuity settings (Alanazi 2024; Alodhialah et al. 2024). The absence of formal support mechanisms, including debriefing and counseling, exacerbates emotional strain and threatens both nurse well-being and retention. Institutional strategies to foster resilience, such as structured peer support, reflective supervision, and targeted mental health resources, are critical to sustaining the nursing workforce in palliative settings.

Spiritual and cultural care is another key dimension of palliative nursing in Saudi Arabia. Nurses frequently provide religious and spiritual support, yet formal training in spiritual care is often lacking (Alshammari et al. 2023; Mani 2024). Structured integration of spiritual care competencies into nursing education and institutional protocols is essential to ensure that care aligns with patient and family beliefs while maintaining professional standards. Collaboration with religious leaders and culturally-informed care frameworks can enhance both patient satisfaction and nurse confidence in delivering spiritual support (Almulla et al. 2024).

Patient and family outcomes were positively influenced by the presence of structured palliative services, including home-based care and multidisciplinary palliative teams (Callaghan et al. 2024; Abdullah et al. 2025). Families reported improved satisfaction, enhanced symptom control, and better care coordination when specialized services were available. However, unequal service distribution created inequities, highlighting the need for expansion of palliative services across underserved regions and for policy frameworks that support consistent, high-quality care delivery (Alshammary et al. 2024).

Systemic and institutional barriers were pervasive, including workforce shortages, lack of standardized care protocols, limited infrastructure for home-based services, and challenges associated with a culturally diverse nursing workforce (Almobarak 2024; Alqahtany et al. 2025). These structural issues impede the consistent delivery of quality care and necessitate coordinated national and institutional strategies to standardize protocols, strengthen workforce capacity, and integrate culturally competent care models (Alrimali and Alreshidi 2024).

## Conclusion

This review demonstrates that nurses are central to palliative and EOL care delivery in Saudi Arabia, yet their effectiveness is limited by gaps in education, communication skills, emotional support, and system-level infrastructure. While structured palliative services improve patient comfort and family satisfaction, access remains uneven across regions. Targeted investments in nursing education, culturally sensitive communication training, emotional well-being programs, and standardized national palliative care frameworks are essential to strengthening nursing practice and ensuring equitable, high-quality EOL care nationwide. Future research should focus on evaluating the effectiveness of educational interventions, developing culturally tailored training programs, and assessing innovative models of palliative nursing practice to optimize patient and family outcomes.

### Recommendations

Based on the findings, several recommendations emerge to improve palliative and EOL nursing care in Saudi Arabia:

**Short-term priorities**:

1. Enhance nursing education and professional development: Integrate comprehensive palliative care modules covering pain and symptom management, ethical decision-making, and spiritual care competencies into undergraduate and postgraduate curricula, complemented by in-service training programs.
2. Implement culturally sensitive communication programs: Equip nurses with skills to navigate family-centered decision-making and ethically complex discussions at the end of life.
3. Provide emotional support for nurses: Establish structured debriefing sessions, counseling services, reflective supervision, and resilience-building programs to reduce emotional burden and prevent burnout.

**Long-term priorities**:

1. Expand and standardize palliative services: Develop national and institutional policies to increase access to home-based and specialized palliative care services, implement standardized care protocols, and strengthen the nursing workforce to ensure equitable care delivery across regions.
2. Integrate spiritual care into nursing practice: Formally embed spiritual support within nursing roles, guided by culturally appropriate frameworks and collaboration with religious leaders, to enhance patient and family well-being.

### Implications

The findings of this review have important implications for nursing practice, education, and policy. For nursing practice, the central role of nurses in providing culturally competent, patient-centered palliative care highlights the need for institutional support in communication, emotional well-being, and integration of spiritual care to enhance care quality and reduce staff burnout. In education, embedding comprehensive palliative care content and practical training into undergraduate and postgraduate nursing programs is essential to strengthen knowledge, skills, and competencies, ensuring nurses are prepared to deliver culturally sensitive EOL care. From a policy perspective, addressing systemic barriers such as workforce shortages, uneven access to services, and the absence of standardized care protocols is critical to improving the quality and equity of palliative care nationwide. Additionally, policy frameworks should incorporate culturally and contextually appropriate guidelines that reflect societal and religious norms, guiding healthcare planning and service provision.

### Limitations

This systematic review has several limitations. First, the included studies were heterogeneous in design, settings, samples, and measurement instruments, which limited direct comparison of findings and precluded meta-analysis. Second, several studies provided limited contextual or regional detail, potentially affecting the generalizability of findings across diverse healthcare settings in Saudi Arabia. Third, the review relied on published literature and accessible grey sources, which may have introduced publication bias by excluding relevant unpublished studies or studies with non-significant results. Similarly, the inclusion of studies published only in English and retrievable Arabic-language sources may have led to language bias, potentially omitting relevant research not indexed in the selected databases. In addition, most included studies focused on adult and critical-care populations, with limited representation of pediatric palliative care, community-based services, or primary care settings, indicating gaps in the current evidence base and limiting understanding of the full spectrum of palliative care needs nationwide. Although selected nonempirical sources (e.g., policy reports and expert commentaries) were included to provide contextual and system-level insights, the primary synthesis and conclusions regarding nursing practice were derived exclusively from empirical studies. Finally, the review protocol was not prospectively registered (e.g., in PROSPERO); however, the review adhered to PRISMA guidelines, applied predefined eligibility criteria, and used independent screening and quality appraisal processes to enhance methodological rigor and transparency.

## Supporting information

10.1017/S147895152610203X.sm001 Ibrahim and Zaghamir supplementary material Ibrahim and Zaghamir supplementary material

## Data Availability

All data analyzed in this review were derived from published studies and publicly available sources. No new datasets were generated.

## Supplementary material

The supplementary material for this article can be found at https://doi.org/10.1017/S147895152610203X.
