# Supplementary material for: Palliative and end-of-life nursing care in Saudi Arabia: A systematic review of nursing practices, challenges, and patient–family outcomes
Source: Palliat Support Care. 2026 Mar 6;24:e82. doi: 10.1017/S147895152610203X (PMC13166443; doi:10.1017/S147895152610203X)
Supplement: Ibrahim and Zaghamir supplementary material [file S147895152610203Xsup001.pdf]

Supplementary File 1: Search Strategy

Title: Example Search Strings and Boolean Combinations Used in the Systematic Review of Palliative and End-of-Life Nursing Care in Saudi Arabia

Purpose: This file provides detailed search strategies, including Boolean operators, keywords, and filters, for each database searched. It enhances transparency and reproducibility of the systematic review.

**Database**	**Search String / Boolean Combination**	**Filters / Limits**	**Notes**
**PubMed**	("palliative care" OR "end-of-life care") AND (nursing OR nurse*) AND ("Saudi Arabia") AND ("home-based care" OR "spiritual care" OR communication OR "symptom management")	Language: English, Arabic; All years	MeSH terms used where applicable; keywords searched in title/abstract
**CINAHL (EBSCO)**	("palliative care" OR "end-of-life care") AND (nursing OR nurse*) AND ("Saudi Arabia") AND ("home-based care" OR "spiritual care" OR communication OR "symptom management")	Language: English, Arabic; All years	CINAHL Headings included when available
**Scopus**	TITLE-ABS-KEY("palliative care" OR "end-of-life care") AND TITLE-ABS-KEY(nursing OR nurse*) AND TITLE-ABS-KEY("Saudi Arabia") AND TITLE-ABS-KEY("home-based care" OR "spiritual care" OR communication OR "symptom management")	Language: English, Arabic; All years	Keywords applied to title, abstract, and author keywords
**Web of Science**	TS=("palliative care" OR "end-of-life care") AND TS=(nursing OR nurse*) AND TS=("Saudi Arabia") AND TS=("home-based care" OR "spiritual care" OR communication OR "symptom management")	Language: English, Arabic; All years	Topic search includes title, abstract, and keywords
**Google Scholar**	"palliative care" OR "end-of-life care" AND nursing AND "Saudi Arabia" AND ("home-based care" OR "spiritual care" OR communication OR "symptom management")	Language: English, Arabic; First 200 results screened	Search strategy adapted for broad coverage of grey and peer-reviewed literature
**Saudi Digital Library / Ministry of Health Reports**	"palliative care" OR "end-of-life care" AND nursing AND "Saudi Arabia"	Language: English, Arabic; All years	Grey literature search to capture theses, institutional reports, and Ministry of Health documents

Notes:

1. Boolean operators AND and OR were applied to combine search concepts effectively.
2. No date restrictions were applied to capture the full historical and current scope of research in Saudi Arabia.
3. Both English and Arabic publications were included to ensure cultural and linguistic representation.
4. The search strategy was intentionally broad to include both academic and practice-oriented literature across diverse healthcare settings, including hospitals, home health care, and academic dissertations.
5. All search results were screened following PRISMA 2020 guidelines, ensuring transparency and reproducibility.
6. Adjustments were made for each database based on its specific search syntax (e.g., MeSH terms in PubMed, subject headings in CINAHL).
